# Neuronally produced betaine acts via a ligand-gated ion channel to control behavioral states

**DOI:** 10.1073/pnas.2201783119

**Published:** 2022-11-21

**Authors:** Iris Hardege, Julia Morud, Jingfang Yu, Tatiana S. Wilson, Frank C. Schroeder, William R. Schafer

**Affiliations:** ^a^MRC Laboratory of Molecular Biology, Cambridge CB2 0QH, United Kingdom; ^b^Boyce Thompson Institute, Cornell University, Ithaca, NY 14853; ^c^Department of Chemistry and Chemical Biology, Cornell University, Ithaca, NY 14853; ^d^Department of Biology, KU Leuven, Leuven 3000, Belgium

**Keywords:** betaine, ion channel, *C. elegans*, behavior

## Abstract

Trimethylglycine, or betaine, is an amino acid derivative found in diverse organisms, from bacteria to plants and animals, with well-established functions as a methyl donor and osmolyte in all cells. In addition, betaine is found in the nervous system, though its function there is not well understood. Here, we show that betaine is synthesized in the nervous system of the nematode worm, *Caenorhabditis elegans,* where it functions in the control of different behavioral states. Specifically, we find that betaine can be produced in a pair of interneurons, the RIMs, and packed into synaptic vesicles by the vesicular monoamine transporter, CAT-1, expressed in these cells. Mutant animals defective in betaine synthesis are unable to control the switch from local to global foraging, a phenotype that can be rescued by restoring betaine specifically to the RIM neurons. These effects on behavior are mediated by a newly identified betaine-gated chloride channel, LGC-41, which is expressed broadly in the navigation circuit. These results implicate neuronally produced betaine as a neuromodulator in vivo and suggest a potentially similar role for betaine in nervous systems of other animals.

## Significance

Controlling food search behavior in changing environments is critical to the survival of all animals. We have found that food search behavior in the nematode *C. elegans* is controlled by betaine, a noncanonical neuromodulator derived from choline. Betaine is synthesized and released from a pair of interneurons and is required to regulate the switch between local and global foraging patterns. We determined that betaine acts through an inhibitory ion channel expressed in diverse neurons in the neural circuits controlling navigation. These results show that betaine, well known for its roles as a methyl donor and osmolyte, can function as a neuromodulator in the nematode nervous system, raising the possibility of a similar role in the human brain.

The amino acid derivative betaine, or trimethylglycine, is a ubiquitous small molecule typically synthesized from the oxidation of choline. In mammals, betaine is best characterized for its role in osmoregulation; for example, in the kidney medulla, betaine transport is essential for adaptation to hypertonic stress (1). Even in unicellular organisms, betaine acts as an osmolyte, with many bacterial species expressing betaine transporters which are critical for maintaining cellular osmolarity (2). In other tissues, for example the mammalian liver, betaine acts as a methyl donor during the transmethylation of homocysteine to methionine (3).

Betaine is present in the brain, and the identification of BGT1 (4), a betaine and GABA-selective transporter with relatively low affinity for GABA (gamma-aminobutyric acid) compared with other GABA transporters (5), has led to a growing interest in the role of betaine in the nervous system. A building body of evidence suggests that betaine may indeed have specific roles in the nervous system; for example, dietary supplementation with betaine has been shown to improve cognitive performance in humans as well as improving memory in rodents (6). In addition, betaine has been shown to have anticonvulsant properties, such as inhibiting pharmacologically induced seizures in rats (7, 8). Moreover, inhibitors of BGT1, expressed in neuronal dendrites, have shown promise as potential antiepileptic drugs (8). Although these effects could be mediated indirectly, for example through effects on local concentrations of GABA or on neuronal metabolism, the effects are also consistent with a direct action of betaine on neurons. However, a molecular target for betaine in mammalian neurons has not yet been identified.

Interestingly, the nematode *Caenorhabditis elegans* (*C. elegans*) expresses a homolog of BGT1, *snf-3,* which encodes a betaine-selective transporter, without measurable activity towards GABA (9). In addition *C. elegans* also expresses a muscular ligand-gated ion channel, *acr-23*, that is activated by betaine (9, 10). These results, like the previously described mammalian evidence, raise the question of whether betaine might have a functional role in the control of normal worm behavior. However, it is not known whether betaine is synthesized or released from nematode neurons or how such release might act to modulate neural circuits.

## LGC-41 Is an Inhibitory Betaine Receptor Expressed Broadly in the Nervous System.

**Fig. 1.**
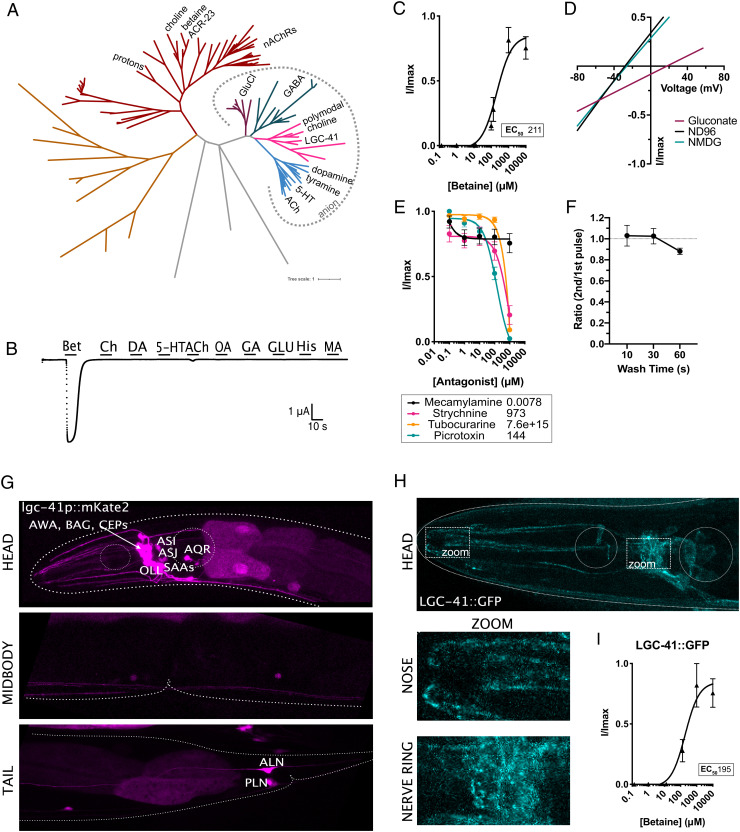
LGC-41 forms a homomeric inhibitory betaine-gated channel. (*A*) Phylogenetic tree showing evolutionary relationship between *C. elegans* LGICs, branch colors show subfamilies as follows: nAChRs (red), uncharacterized (orange and grey), GluCl (purple), GABA-gated anion and cation channels (green), diverse channels including LGC-41 (pink), ACh-gated and monoamine-gated channels (blue). Figure made with iTOL using an unrooted tree with one iteration of the equal-daylight algorithm (*B*) Continuous current trace for an oocyte expressing LGC-41 held at −60 mV and perfused with a panel of ligands. (*C*) Dose–response curve for oocytes expressing LGC-41 exposed to increasing concentrations of betaine, error bars represent SEM of at least five oocytes. *Inset* shows EC_50_ in μM calculated with a three parameter Hill slope. (*D*) Representative plot of current-voltage relationship of betaine-induced current in oocytes expressing LGC-41 in ND96 (Na^+^ & Cl^−^ present), Na Gluconate (low Cl^−^) and NMDG (no Na^+^), mean ΔErev for ND96 vs. Na Gluconate: −43.1 mV, SEM: 0.8 mV, N = 9 oocytes. (*E*) Antagonist dose–response curves in oocytes expressing LGC-41 in the presence of 500 μM betaine and increasing concentrations of each antagonist, error bars represent SEM of at least seven oocytes. *Inset* shows IC_50_ in μM calculated with a three parameter Hill slope. (*F*) Ratio of betaine-induced current in oocytes expressing LGC-41 exposed to multiple pulses of 500 μM betaine with 10-, 30-, and 60-s wash time between pulses. Error bars represent SEM of at least 6 oocytes. (*G*) Fluorescent reporter of mKate2 driven under the promoter sequence of *lgc-41*. (*H*) Images of LGC-41::GFP tagged endogenously with CRISPR/Cas9 in the M3/4 loop, dotted boxes indicate zoomed areas which show localization in the nose and nerve ring. (*I*) Dose–response curve for oocytes expressing LGC-41::GFP exposed to increasing concentrations of betaine, error bars represent SEM of at least four oocytes. *Inset* shows EC_50_ in μM calculated with a three parameter Hill slope.

We identified a putative neuronal receptor for betaine in a survey of orphan ligand-gated ion channels (LGICs) from *C. elegans*. In comparison with mammals, the *C. elegans* genome contains an expanded number of LGIC genes (11), many without identified ligands. By expressing orphan channels in *Xenopus* oocytes and screening for induction of current in the response to a panel of potential ligands, we identified a betaine-gated ion channel, LGC-41 (Fig. 1 *A* and *B*). LGC-41 belongs to a diverse gene family of LGICs in *C. elegans*, whose other members include choline, acetylcholine, or monoamine-gated chloride channels (12) (Fig. 1*A*). Despite its closest human homologues being the glycine receptors (38% identity to GlyR a2, *SI Appendix*, Fig. S1), of the panel of neurotransmitters we tested, LGC-41 was specifically gated only by betaine (Fig. 1 *A* and *B*), with an EC_50_ of 211 μM (Fig. 1*C*), yet we saw no large activation  in the presence of acetylcholine, choline, or any of the other tested neurotransmitters (Fig. 1*B*). An EC_50_ for betaine in this range is in line with the primary agonist of previously studied members of this family of *C. elegans* channels (13–16), as well as related receptors such as mammalian GABA_A_ receptors (17), when expressed in *Xenopus* oocytes. Using ion substitution experiments, we determined that LGC-41 is an inhibitory chloride channel, with a mean ΔErev of −43 mV when Cl^−^-containing extracellular solution is replaced with a low Cl^−^ solution, in line with the rest of this subfamily (Fig. 1*D*). LGC-41 was not effectively blocked by the cholinergic blockers, mecamylamine and tubocurarine, at low concentrations (Fig. 1*E*). Only strychnine, traditionally considered a glycine receptor antagonist, was able to block LGC-41 with an IC_50_ of 973 μM (Fig. 1*E*). This IC_50_, approximately two orders of magnitude higher than for glycine receptors (18), and one order of magnitude higher than for mammalian alpha 7 nAChRs (19), suggesting weak inhibition of betaine-induced current by strychnine. Interestingly, LGC-41 could be blocked by the anion pore blocker picrotoxin with an IC_50_ of 144 μM (Fig. 1*E*). When exposed to multiple applications of betaine, we saw no reduction in the peak current size between pulses of 10-s, 30-s, or 60-s intervals, suggesting that the receptor is accessible for reactivation already after 10 s (Fig. 1*F*).

To determine the function of LGC-41 within the nervous system, we first characterized the expression of the channel using fluorescent reporters under the control of the *lgc-41* promoter. This revealed broad expression of *lgc-41* in several neuronal classes including the sensory neurons AWA, BAG, CEP, AQR, OLL, ASJ, and ASI (Fig. 1*G*). Many of these neurons are known to be involved in regulating behavioral responses that occur during changing environments; AQR and BAG regulate oxygen sensation and social feeding (20), the CEPs induce behavioral changes in response to food sensation (21), and ASI is involved in integrating multiple sensory inputs to regulate feeding and food search behaviors (22) (Fig. 1*G*). To assess the intracellular localization of LGC-41, we generated a single-copy functional green fluorescence protein (GFP)-tagged LGC-41, integrated at the chromosomal locus by CRISPR insertion, which when expressed in *Xenopus* oocytes appeared to form a functional channel with a comparable betaine EC_50_ to the wild-type channel (Fig. 1*I*). We observed localization of LGC-41::GFP in clear punctate structures in the synaptically dense nerve ring region as well as more diffuse staining including in the vicinity of sensory nerve endings in the nose (Fig. 1*H*). This localization suggests a possible neuronal function for LGC-41 and its ligand, betaine, in neuronal communication or chemosensation.

## LGC-41 Promotes Global Search Behavior.

**Fig. 2.**
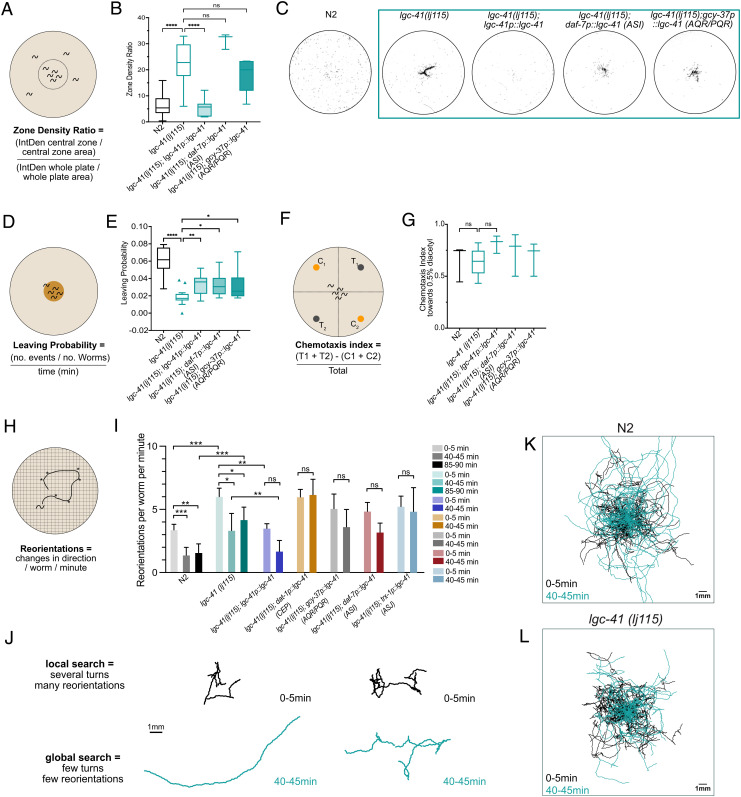
LGC-41 is required for global search and food leaving behaviors. Behavioral responses of N2 (wild-type), *lgc-41(lj115)lgc-41(lj115); lgc-41p::lgc-41, lgc-41(lj115); daf-7p(ASI)::lgc-41, lgc-41(lj115); gcy-37p(AQR/PQR)::lgc-41*. (*A*) Schematic representation of the experimental design and (*B*) Tukey’s box and whisker plot of dispersal assay, in which the zone density ratio of the central zone vs. the whole plate is calculated and plotted. N = 3–29 plates per genotype. (*C*) Representative images of threshold-filtered plates used to calculate dispersal index. (*D*) Schematic representation of the experimental design and (*E*) box and whisker plot of food leaving probability 6 h after being placed on the food patch. N = 16–21 plates per genotype. (*F*) Schematic representation of experiment (*G*) Chemotaxis index toward 0.5% diacetyl. N = 3–5 plates per genotype presented in a box and whisker plot. (*H*) Schematic representation of the experimental design. (*I*) Amount of reorientation events off food at three different time points, as measured per worm per minute. N = 99–157 per genotype. (*J*) Representative trajectories of individual N2 or *lgc-41* mutant worms at 0–5 min and 40–45 min after removal from a food patch. (*K* and *L*) Overlaid trajectories of individual N2 or *lgc-41* mutant worms at 0–5 min and 40–45 min after removal from a food patch. (*K*) 0–5 min: N = 95, 40–45 min: N = 85 individual worms. (*L*) 0–5 min: N = 58, 40–45 min: N = 52 individual worms. (*B*, *C*, *E*, and *G*) Tukey’s boxplots showing the median and one-way ANOVA with Bonferroni (*B* and *G*) or Tukey’s (*C* and *E*) correction for multiple comparisons, *P < 0.05, **P < 0.005, ****P < 0.0001. (*I*) Bars represent the median value with 95% CI and significance test using the Kruskal–Wallis test *P < 0.05, **P < 0.005, ***P < 0.0005.

To examine the role of LGC-41 in the *C. elegans* nervous system, we characterized the behavior of *lgc-41* null mutant animals. In the presence of food, we observed a small but significant decrease in forward speed of *lgc-41* mutant animals and in the fraction of time worms moved forward; however, backward locomotion was not altered (*SI Appendix*, Fig. S2 *E*–*H*). Interestingly, supplementation of the bacteria with betaine was unable to rescue these phenotypes (*SI Appendix*, Fig. S2 *E–H*), consistent with LGC-41 acting as a betaine receptor in vivo. However, the most striking phenotype we observed was that in the absence of food, *lgc-41* mutant animals remained in the center of a plate in large numbers, in stark contrast to the typical dispersal and search behaviors initiated by wild-type worms in the absence of food. Normally, *C. elegans* transition between two distinct search behaviors in the absence of food: the first, local search or area restricted search, lasts approximately 15 min and consists of frequent turns and short reversals, while the second, global search or dispersal, is characterized by long forward runs (22, 23). Reasoning that the lack of dispersal from the center of the plate indicated a change in this search state transition, we systemically examined whether *lgc-41* mutant worms were able to perform the switch between the two search behaviors, local and global search. We placed a high density of washed worms in the center of an empty plate containing no bacteria and observed whether they dispersed to explore the area of the plate after 1 h (Fig. 2*A*). Indeed, we found that *lgc-41* mutants were significantly less likely to disperse across the plate than the wild-type control (Fig. 2 *B* and *C*). To understand the dynamics of this food-search phenotype, we investigated the propensity of *lgc-41* mutant animals to leave a small food patch over time (Fig. 2*D*). The probability of worms to leave a food patch was measured at several time points after the animals were placed onto the assay plates, from 1 h to 9 h. At the timepoints 6 h and 9 h, we observed a significantly lower probability of leaving events for *lgc-41* mutant animals compared with N2 (Fig. 2*E* and *SI Appendix*, Fig. S3*A*). Due to these results, only the 6-h timepoint was used in further experiments.

To understand if the differences observed in the food leaving probability, or dispersal, could be due to defects in basal locomotion off food or chemotaxis defects, we assessed these behaviors in *lgc-41* mutants. In the absence of food, *lgc-41* mutants displayed no significant differences in forward or reverse locomotion compared with wild type (*SI Appendix*, Fig. S2 *A*–*D*), suggesting that their locomotion was grossly normal under these conditions. In addition, the ability to chemotax towards diacetyl, a known chemoattractive odor (24), was not significantly impaired in the *lgc-41* mutant (Fig. 2 *F* and *G*). A worm’s decision to leave a patch of food is dependent on the integration of sensory inputs into the second-order interneurons, such as RIM (22); we therefore tested whether LGC-41 was required for detecting the aversive odor 2-nonanone in the absence or presence of food (25, 26). We saw no reduction in the ability of *lgc-41* mutants to detect 2-nonanone off food; indeed, they showed an increased aversion to 2-nonanone compared with wild-type (*SI Appendix*, Fig. S3*D*). Despite this increased aversion to 2-nonanone, *lgc-41* mutant worms took significantly longer to leave a food patch in the presence of 2-nonaone compared with control animals (*SI Appendix*, Fig. S3 *E*–*H*). By reintroducing a wild-type copy of *lgc-41* under its own promoter, we were able to significantly rescue the behavioral defects observed in the *lgc-41* mutant animals (Fig. 2 *B*–*G* and *SI Appendix*, Fig. S3 *D*, *F*–*H*). Animals expressing the rescue construct under the *lgc-41* promoter had an increased dispersal rate in the absence of food, increased food leaving probability, and increased 2-nonanone-induced food leaving rate. This strongly implicates LGC-41 in the control of food-related behavioral states.

To further assess whether the observed phenotypes were due to a lack of global search initiation, we tracked the behavior of individual animals during the first 5 min after food removal (when animals normally carry out local search), as well as 40–45 min and 85–90 min after food removal (when animals normally perform global search) (Fig. 2 *H*–*L*). Interestingly, during the local search phase (0–5 min) *lgc-41* mutant animals performed significantly more reorientations per minute compared with wild-type animals, suggesting that *lgc-41* mutants are not defective in search behaviors per se (Fig. 2*I*). However, although we observed a clear decrease in turning parameters in wild-type animals during the global search phase (40–45 min and 85–90 min), we did not observe this in *lgc-41* mutants, which appeared to remain in a local search state after food removal, with a significantly higher number of reorientations (Fig. 2*I*) than wild-type. Thus, LGC-41 appears to be required for the transition between the behavioral states underlying local and global food search. By reintroducing *lgc-41* under its own promotor, we were able to rescue the initiation of global food search behavior at the 40–45-min time point (Fig. 2*I*).

LGC-41 is expressed in many neurons implicated in food modulation and navigation behaviors (20–22). To determine which specific *lgc-41*-expressing neurons act to control these behaviors, we expressed a wild-type copy of *lgc-41* under the control of promoters expressed in ASI (*daf-7*), AQR/PQR (*gcy-37*), CEP (*dat-1*), and ASJ (*trx-1*) and tested for phenotypic rescue. Interestingly, transgenic expression of *lgc-41* in both ASI and AQR/PQR, led to small but significant increases in food leaving probability (Fig. 2*E*), though they did not significantly rescue the dispersal behavior (Fig. 2*B*), nor did transgenic expression in CEPs or ASJ (*SI Appendix*, Fig. S3*B*). None of the cell-specific recue strains tested were able to fully recapitulate the global food search behavior (Fig. 2*I*). Furthermore, the 2-nonanone-induced food leaving behaviors could also not be rescued by AQR/PQR expression (*SI Appendix*, Fig. S3 *F* and *G*). These results suggest the expression of *lgc-41* is required in several neuronal classes to control foraging behaviors.

## Betaine Is Synthesized in Specific Interneurons That Control Behavioral States.

**Fig. 3.**
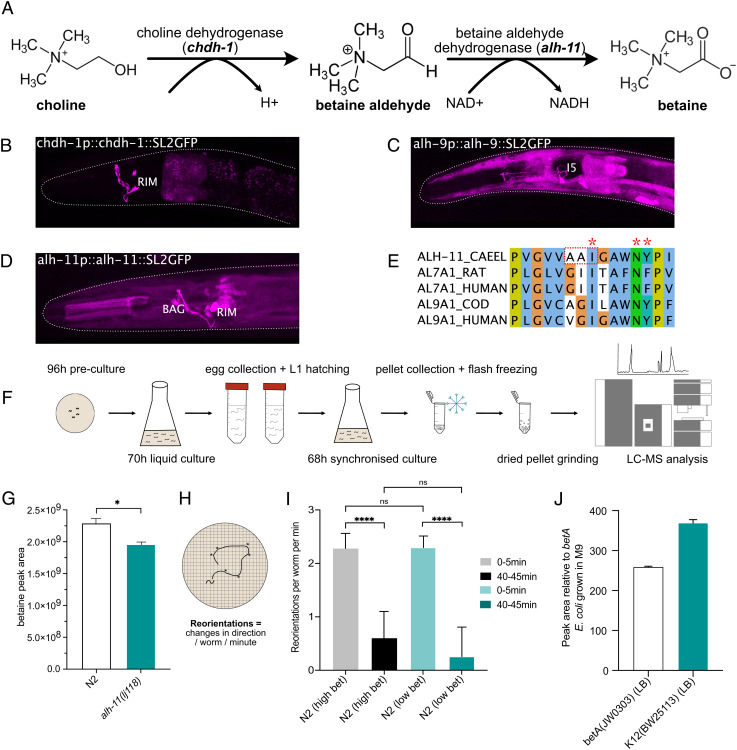
Neuronal *C. elegans* betaine synthesis pathway involves *alh-11*. (*A*) Schematic of the betaine biosynthesis pathway and putative enzymes involved in each stage. (*B–D*) Fluorescent reporter of intercistonically spliced GFP driven under the promoter and genomic sequence of the putative betaine synthesis enzymes *chdh-1, alh-9, and alh-11*. (*E*) Cropped alignment of the substrate-binding region of *C. elegans* ALH-11 and related genes from rat, human and cod. Stars highlight conserved residues required for substrate and NAD^+^ binding as described by ref. 27, residues within the red box are mutated in *alh-11(lj118)* from AAI to --V, the full alignment can be found in *SI Appendix*, Fig. S1*B*. (*F*) Schematic describing the experimental procedure for metabolite extraction paired with LC–MS analysis in *C. elegans* preparations. (*G*) Mass spectrometry analysis of betaine content in *C. elegans* wild-type or *alh-11* mutant preparation. The data show peak area for betaine. Error bars represent SEM of three samples per genotype, *P < 0.05 calculated by unpaired *t*-test. (*H*) Schematic representing the experimental design. (*I*) Food search behavior as calculated by reorientations per worm per minute in N2 worms grown under betaine-deficient conditions using the strain *betA(JW0303)*, grown in M9 media, or betaine-containing Lennox Broth (LB) media. N = 75–133 worms per condition. Bar charts showing median with 95% CI and significance test using the Kruskal–Wallis test *P < 0.05, **P < 0.005, ***P < 0.0005. (*J*) Relative betaine amount detected using quantitative mass spectrometry in the *betA(JW0303)* and K12(BW25113) strains grown in LB media to the *betA* grown in M9 media.

The involvement of a betaine receptor in the control of navigation states raised the question of what role betaine might play in the modulation of these behaviors. Since GFP-tagged LGC-41 protein appeared to be localized both in the nerve ring, the main synaptic neuropil of the worm, as well as in sensory processes, we considered two hypotheses: exogenous betaine might function as a chemosensory cue, for example to indicate the presence of bacteria, or alternatively endogenous betaine could function as a neuromodulator to directly modulate neural states. We first tested if wild-type worms could sense exogenous betaine. We observed no positive or negative chemotaxis toward betaine in either wild-type or *lgc-41* mutant worms (*SI Appendix*, Fig. S4 *A* and *B*) suggesting that betaine might not be an important chemosensory cue. Alternatively, to investigate whether betaine might be produced endogenously in the nervous system, we investigated the expression patterns of the putative betaine synthesis pathway genes *alh-9, alh-11,* and *chdh-1* (Fig. 3 *A* and *E* and *SI Appendix*, Fig. S1*B*). The genes *alh-9* and *alh-11* were selected as putative betaine aldehyde dehydrogenases based upon their homology with ALDH7A1 (human betaine aldehyde dehydrogenase), with 65% and 27% identity, respectively. *chdh-1* was selected as a putative choline dehydrogenase due to its 55% identity to CHDH (human choline dehydrogenase) (*SI Appendix*, Fig. S1*B*). By characterizing the expression patterns of transgenic reporters, we identified expression of all three putative biosynthesis pathway genes in neurons (Fig. 3 *B*–*D*). In particular, the RIM neurons showed expression of both *chdh-1* and *alh-11*, indicating that these neurons have the capacity to synthesize betaine from choline.

To test whether one of these genes, *alh-11,* the betaine aldehyde dehydrogenase homolog expressed in RIM, is involved in synthesizing betaine *in vivo*, we generated animals carrying a mutation in the conserved substrate-binding site of *alh-11* (Fig. 3*E*) (27), then performed quantitative mass spectrometry analysis of wild-type and *alh-11* mutant worms (Fig. 3 *F* and *G*). In both samples, high levels of betaine could be detected; however, there was a significant reduction in betaine levels in *alh-11* mutant worms compared with control worms (Fig. 3*G*). This strongly supports the notion that betaine is produced endogenously within the worm, and that ALH-11 is required for betaine synthesis in at least some tissues. The remainder of the betaine detected in *alh-11* mutant animals may be a result of residual activity by the mutant form of ALH-11*,* contribution by ALH-9 or resulting from the bacterial food source, which contains high levels of betaine (*SI Appendix*, Fig. S4*G*). We were unable to generate null mutations for *alh-9*, *alh-11,* or *chdh-1* as these resulted in lethality. To further address if exogenous betaine could influence food search behavior in worms, we tested the ability of wild-type N2 worms to perform local and global search after being fed with the betaine-deficient *Escherichia coli* strain, *betA(JW0303)* (Fig. 3 *H* and *I*). We compared the behavior of worms fed with *betA* bacteria cultured in minimal M9 media, and worms fed on the *betA* bacteria grown in normal LB media containing betaine. We did not detect any differences in behavior at any time point between the two growth conditions, suggesting the functions of exogenous and neuronal betaine are different. To validate the amount of betaine produced by the *betA* bacteria, as well as the parental *K12(BW25113)* strain, grown in M9, or LB, quantitative mass spectrometry was performed. Indeed, the *betA* strain grown in M9 media contained a hundred-fold less betaine compared with the same bacteria or the parental strain K12 grown in LB (Fig. 3*J*).

**Fig. 4.**
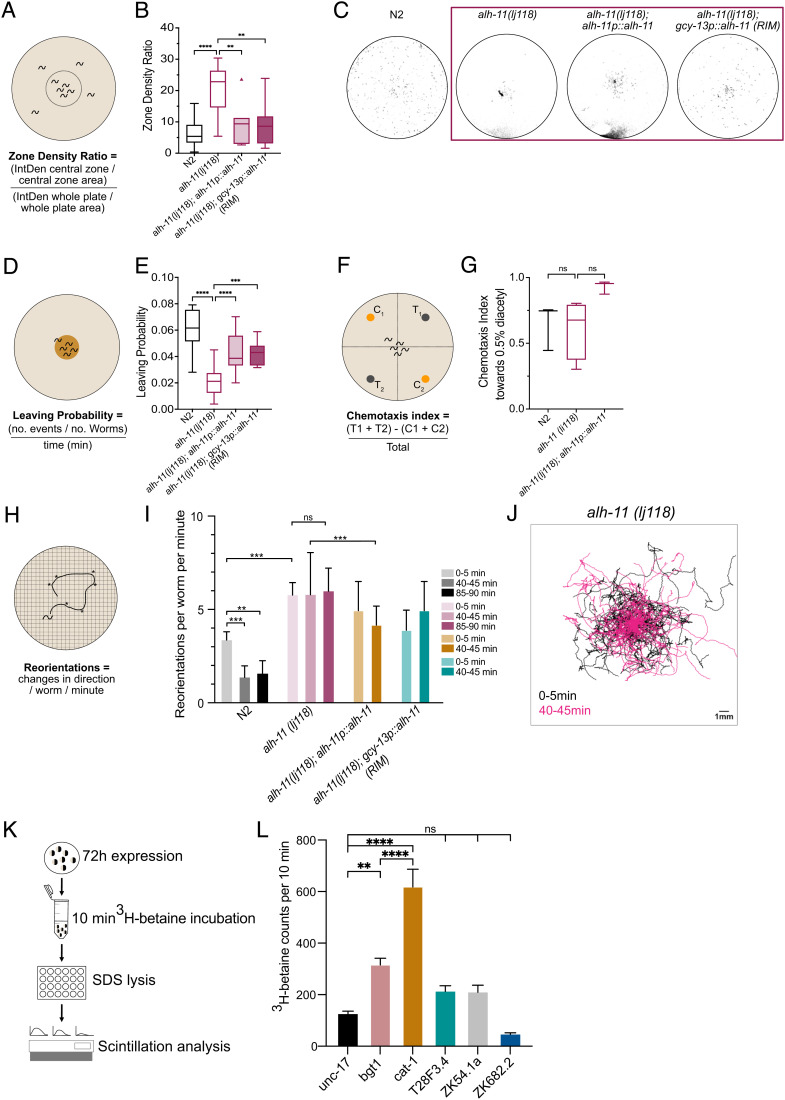
Endogenously produced betaine is required to promote global search and food leaving behaviors. Behavioral responses of N2 (wild-type), *alh-11(lj118), alh-11(lj118); alh-11p::alh-11,* and *alh-11(lj118); gcy-13p(RIM)::alh-11* worms. (*A*) Schematic representation of the experimental design and (*B*) Tukey’s boxplot of dispersal assay, in which the zone density ratio of the central zone vs. the whole plate is calculated and plotted. N = 3–29 plates per genotype. (*C*) Representative images of threshold-filtered plates used to calculated dispersal index. (*D*) Schematic representation of the experimental design and (*E*) Tukey’s box plot of food leaving probability 6 h after being placed on the food patch. N = 16–21 plates per genotype. (*F*) Schematic representation of the experiment (*G*) Chemotaxis index toward 0.5% diacetyl. N = 3–5 plates per genotype presented in a box and whisker plot. (*H*) Schematic representation of the experimental design (*I*) Amount of reorientation events off food at three different time points, as measured per worm per minute. N = 80–157 per genotype. Bars show median value with 95% CI and significance test using the Kruskal–Wallis test *P < 0.05, **P < 0.005, ***P < 0.0005. (*J*) Overlaid trajectories of individual *alh-11* mutant animals at 0–5 min and 40–45 min after removal from a food patch. 0–5 min: N = 69, 40–45 min: N = 109 individual worms. (*K*) Schematic outlining workflow for transporter assay in *Xenopus* oocytes. (*L*) ^3^H-betaine uptake during 10 min evaluated in the *C. elegans* genes *unc-17, cat-1, T28F3.4, ZK54.1a,* and *ZK682.2* as well as the human betaine/GABA transporter *BGT1* (SLC6A12). Error bars represent SEM of 6–10 samples per genotype, **P < 0.01, ****P < 0.0001, calculated by a one-way ANOVA using Sidak’s test for multiple comparisons. (*B*, *C*, *E*, and *G*) Tukey’s boxplots showing the median value and a one-way ANOVA with Bonferroni (*B* and *G*) or Tukey’s (*C* and *E*) correction for multiple comparisons, **P < 0.005, ***P < 0.001, ****P < 0.0001.

We next sought to understand if endogenously produced betaine might act *in vivo* to control the behaviors requiring the betaine receptor LGC-41. We therefore characterized the behavior of *alh-11* mutant animals, which have reduced endogenous betaine levels (Fig. 3*G*). First, we noted that like the *lgc-41* null mutant animals, in the presence of food, *alh-11* mutant animals showed a reduced fraction of forward locomotion and reduced forward speed (*SI Appendix*, Fig. S2 *E*–*H*). However, in contrast to the phenotype induced by loss of the betaine receptor *lgc-41*, the reduction in forward speed seen in *alh-11* mutant animals could be rescued by supplementing with exogenous betaine (*SI Appendix*, Fig. S2*H*), in line with our mass spectrometry findings, which showed that these animals produce less betaine. In addition, we observed more strikingly similar behavioral defects in the *alh-11* mutants compared with *lgc-41* mutants. Like the animals lacking the betaine receptor *lgc-41*, *alh-11* mutant animals had a reduced ability to disperse in the absence of food (Fig. 4 *A*–*C*), a reduced propensity to leave a food patch over time (Fig. 4 *D* and *E*) and appeared to remain in the local search state 40–45 min after removal from food (Fig. 4 *H*–*K*). Interestingly, unlike the receptor mutants, *alh-11* mutants were not defective in 2-nonanone-induced food leaving (*SI Appendix*, Fig. S4 *D*–*F*), suggesting other betaine sources may be relevant, for example, bacterial betaine production (Fig. S4*G*) or biosynthesis via *alh-9*, for which we were unable to generate null alleles due to homozygote lethality. Instead, we generated a cre-inducible *alh-9* CRISPR knockout line, and by expressing cre in the RIM neurons, attempted to knock out *alh-9* specifically in RIM. However, we were unable to detect any defects in food search behavior in these animals (Fig. S5*C*). Like *lgc-41* mutants, *alh-11* mutants showed few significant differences in basal locomotion in the absence of food (*SI Appendix*, Fig. S2 *A*–*D*) and no defects in chemotaxis (Fig. 4 *F* and *G* and *SI Appendix*, Fig. S4*C*). Moreover, all the food search phenotypes were rescued by transgenic expression of the *alh-11* gene under its own promoter (Fig. 4 *B*–*E*). These results indicate that betaine, synthesized endogenously through the action of ALH-11, is involved in modulating the switch between local and global search behaviors.

Since *alh-11* is expressed in neurons, we hypothesized that betaine might be released from specific neurons to control behavioral states. In particular, both *alh-11* and *chdh-1*, which encodes the other enzyme required to synthesize betaine from choline, are expressed in the RIM neurons, which have been strongly implicated in food-related behaviors and decision-making (28). To test whether the RIMs might release betaine as a neuromodulator, we tested whether restoring ALH-11 activity in these neurons was sufficient to rescue the *alh-11* food search phenotypes. Indeed, when we reintroduced a rescuing copy of *alh-11* under the control of a RIM-specific promoter (*gcy-13*) (*SI Appendix*, Fig. S4*H*), this significantly rescued the dispersal defect in the absence of food (Fig. 4 *B* and *C*) as well as the food leaving phenotype on a small food patch (Fig. 4*E*). These results indicate that betaine release from RIM alone is sufficient to support wild-type food search behavior and provides *in vivo* evidence that neuronally produced betaine functions as a neuromodulator.

These results raised the question of how betaine might be released from the RIM neurons to control behavior. To investigate the possibility that betaine is loaded into synaptic vesicles, we assayed several vesicular transporters for their ability to transport betaine. We expressed several potential transporters in *Xenopus* oocytes, including two previously characterized *C. elegans* vesicular transporters (*unc-17* and *cat-1*), three uncharacterized solute carrier genes from *C. elegans (T28F3.4, ZK54.1a, ZK682.2)*, and the human betaine/GABA co-transporter *BGT1* (SLC6A12) (Fig. 4 *K* and *L*). Surprisingly, we found that CAT-1, previously described as a vesicular monoamine transporter (29), was also able to transport betaine in high quantities (Fig. 4*L*). Oocytes expressing CAT-1 transported significantly more betaine than the acetylcholine vesicular transporter UNC-17, and even more than the mammalian betaine/GABA transporter BGT1. None of the other *C. elegans* orphan transporters showed similar betaine transport activity (Fig. 4*L*). This suggests that *C. elegans* may have the ability to package betaine in synaptic vesicles via CAT-1. Significantly, CAT-1 is known to be expressed in the RIM neurons (29), where it functions to load tyramine into vesicles. These results indicate that the RIM neurons may therefore co-release tyramine and betaine to control different behaviors. To assess the role for CAT-1 in the switch between local and global food search, we tracked *cat-1* mutant animals and analyzed the number of reorientations animals made per minute at timepoints 0–5 min and 40–45 min after food removal. We found that *cat-1* mutant animals were defective in switching between local and global food search; however, this behavior could not be rescued by reintroducing *cat-1* in RIM using the *gcy-13* promotor (*SI Appendix*, Fig. S5 *A* and *B*).

## Discussion

In this study, we identified a role for a nonclassical neurotransmitter, betaine, and its receptor LGC-41 in the control of behavior. We have found that animals either lacking the betaine receptor, *lgc-41*, or the betaine synthesis gene, *alh-11*, displayed defects in multiple food search behaviors, including food leaving from a limited food source and in the transition from local to global search in the absence of food. These behavioral defects could be rescued by reintroducing the betaine synthesis gene, *alh-11,* specifically in the RIM interneurons, which have been implicated in the integration and decision-making processes during food search behavior (28, 30). Interestingly, we found that the vesicular transporter CAT-1, known to be highly expressed in the RIM interneurons (29), was capable of betaine transport, indicating that these neurons are capable of loading betaine into synaptic vesicles. These results indicate that betaine, released from synaptic vesicles in the RIM neurons, and acting on LGC-41, functions as a neuromodulator to control behavioral states involved with foraging and navigation. The broad expression pattern of LGC-41, covering several neurons known to be involved in food sensation and food search behaviors offers a possible mechanism for changes in global behavioral state upon the release of betaine (*SI Appendix*, Fig. S4*I*).

While *alh-11* defects could be rescued in a single neuron class, the *lgc-41* receptor was expressed broadly in the nervous system, and cell-specific expression in specific neuron classes resulted in only partial rescue. Moreover, while the neuronal projections of LGC-41-expressing neurons all project into the nerve ring, none of these neurons receive significant synaptic input from RIM. These findings, along with the mixture of punctate and diffuse localization of LGC-41 within neurons, suggests that betaine may act extrasynaptically to activate LGC-41 receptors and thereby control behavior. This is consistent with the fact that tyramine, another neuromodulator released from the RIM neurons (31), also appears to function mostly extrasynaptically (32). Assessing the effect of betaine on LGC-41-expressing neurons, using neuronal imaging will be of interest in the future and may uncover the molecular- and circuit-level mechanisms by which betaine acts to control food search behavior.

Previous work has identified another betaine-activated LGIC, the muscular cationic channel ACR-23 (9, 10). Interestingly, ACR-23 is quite distantly related to LGC-41; whereas ACR-23 is related to nAChRs, LGC-41 is more closely related to GABA_A_ and glycine receptors. The presence of two independently evolved betaine-gated channels, along with the presence of a betaine reuptake transporter, *snf-3* (9), suggests a much broader role for betaine in the regulation of worm behaviors than previously recognized. The source of betaine acting via these two opposing receptors is not yet clear. Our study suggests that neuronally produced betaine acts via LGC-41 in at least some neurons. However, worms lacking *alh-11*, the betaine synthesis enzyme, still contained some betaine, and did not display any locomotion defects seen in *acr-23* mutant animals. Thus, worms may contain other sources of betaine which act upon ACR-23 receptors in the muscle, such as a bacterial source. In particular, the other *C. elegans* aldehyde dehydrogenase gene, *alh-9*, is also expressed in neurons, and may be responsible for synthesizing the betaine present in *alh-11* mutants. In addition, several types of bacteria, including *E. coli*, produce betaine to regulate their osmolarity (33), and as such it is possible that betaine intake through food may have behavioral effects, paralleling recent observations concerning ingested octopamine in worms (34).

This work also raises the question of whether betaine might function as a neuromodulator in other animals, including humans. In mammals, betaine transport is closely associated with the transport of GABA (4); thus it is possible that betaine might be cotransported into GABA-containing vesicles, as we hypothesize for tyramine and betaine in *C. elegans*. In rodents, a lack of betaine has been shown to affect the balance of GABAergic transmission, causing changes in memory formation (35). Interestingly, there have also been reports of the upregulation of betaine/GABA transporter expression in rodents after performing learning assays, further indicating a specific role for betaine in the nervous system separate from its osmoregulatory function. Many questions regarding the extent of betaine’s actions within animal nervous systems remain; for example, it is not clear whether betaine can be released from synaptic vesicles in an activity-dependent manner and if so, what conditions and molecular mechanisms might underlie such a process. In the future, it will be interesting to determine whether betaine’s role as a neuromodulator may extend beyond nematodes into other animals.

## Methods

### *C. elegans* Culture.

Unless otherwise specified, *C. elegans* worms were cultured on NGM agar plates with OP50 (36). A full list of strains used in this study can be found in *SI Appendix*, Table S1. Unless otherwise specified 1-d adult worms were synchronized by placing L4 worms on culture plates overnight.

For metabolite extraction experiments, the worms were cultured as follows: Ten *C. elegans* hermaphrodites were placed onto 6-cm NGM plates. Each plate was seeded with 200 μL OP50 *E. coli* grown to stationary phase in LB and incubated at 22°C. After 96 h, each plate was washed with 25 mL S-complete medium into a 125 mL Erlenmeyer flask after which 1 mL OP50 *E. coli* suspension was added to the liquid cultures, shaking at 220 RPM 22°C (*E. coli* cultures were grown to stationary phase in Terrific Broth, pelleted and resuspended at 1-g wet mass per 1 mL M9 buffer). After 70 h, 6 mL bleach was added to each culture, along with 900 μL 10 M NaOH, and the mixture was shaken for 3 min to prepare eggs. Eggs were centrifuged at 1,500× *g* for 30 s, supernatant discarded, and washed with 25 mL M9 buffer three times. The pellet was resuspended in final volume 5 mL M9 and transferred to a new 15 mL centrifuge tube. Eggs were placed on a rocker and allowed to hatch as L1 larvae for 24 h at 22°C. 100,000 L1s were counted and cultured in 25 mL cultures of S-complete with 1 mL of OP50 and incubated at 220 RPM 22°C for 68 h. Worms were then spun at 2,000× *g* for 5 min, and the spent medium was separated from the worm body pellet. The separated medium and worm pellet were flash frozen in liquid nitrogen and stored at −20°C freezer until further processing.

### *Xenopus laevis* Oocytes.

Defolliculated oocytes from *X. laevis* were obtained from EcoCyte Bioscience (Dortmund, Germany) and kept in ND96 (in mM: 96 NaCl, 1 MgCl_2_, 5 HEPES, 1.8 CaCl_2_, 2 KCl) solution at 16°C for 3–5 d after cRNA injection.

### Molecular Biology.

The *lgc-41* cDNA sequence, as well as several vesicular transporters, from wild-type N2 *C. elegans* were cloned from worm cDNA (generated by reverse transcription PCR from total worm RNA using Q5 polymerase (New England Biosciences)). Ion channel, or vesicular transporter constructs for expression in *Xenopus* oocytes, were made from cDNA sequences and cloned into the KSM vector, the gene was inserted between *Xenopus* β-globin 5′ and 3′UTR regions and downstream of a T3 promoter using the HiFi assembly protocol (New England Biosciences). This protocol was also used for generating *C. elegans* expression constructs using the pDESTR4R3II vector. For fluorescent expression, *C. elegans* wild-type N2 gDNA was used for cloning gDNA sequences and the inclusion of GFP or mKate2 on the same plasmid after an intercistronic splice site (SL2 site). Unless otherwise specified, promoter sequences consist of approximately 2 kb gDNA upstream of the start codon.

### CRISPR/CAS9-Mediated Gene Manipulation.

Endogenous tagging of the M3/4 cytosolic loop of LGC-41 with GFP was carried out using the SapTrap protocol (37, 38) for *lgc-41(lj119)*. *C. elegans* genetic modifications of *lgc-41(lj115)* and *alh-11(lj118)* were made as previously described here (39). We also attempted to generate mutations for *alh-9* and *chdh-1* following the same protocol; however, we had no success generating mutations, with many dead animals on the plates and animals lacking gonads, we therefore inferred that these genes were likely to be essential for viability. *lgc-41(lj115)* resulted in a frameshift, whereas *alh-11(lj118)* resulted in a complex mutation with deletion of two amino acids and replacement of one further amino acid in the conserved substrate-binding site (Fig. 3*E* and *SI Appendix*, Fig. S1*B* and Table S2), resulting in 351-353AAI to A351del, A352del and I353V, abolishing the conserved Ile, which is involved in NAD^+^ binding, and potentially disrupting the downstream Asn and Tyr residues which are required for substrate binding (27). *alh-9(syb5001, syb4967)* was generated by SUNY biotechnologies and resulted in the genomic *alh-9* sequenced flanked by 5′ and 3′ *loxP* sites, with inducible expression of mCherry upon successful cre recombination.

### RNA Synthesis and Microinjection.

The T3 mMessage mMachine transcription kit was used for in vitro synthesis of 5′ capped cRNA, the procedure was done according to the manufacturer's protocol (ThermoFischer Scientific). The cRNA was purified prior to injection using the GeneJET RNA purification kit (Thermo Fischer Scientific). *Xenopus* oocytes (Ecocyte) were individually placed into 96-well plates and injected with 50 nL of 500 ng/μL RNA using the Roboinject system (Multi Channel Systems GmbH) or manually injected using a Nanoject II (Drummond Scientific). A 1:1 ratio was used when two constructs were coinjected and with a total 500 ng/μL RNA concentration. The injected oocytes were incubated for 3–6 d post injection at 16°C in ND96 until the day of experiments.

### Two-Electrode Voltage-Clamp (TEVC) Recording and Data Analysis.

Using either the Robocyte2 system or a manual set up with an OC-725D amplifier (Multi Channel Systems, GmbH), two-electrode voltage clamp recordings were carried out. Unless otherwise stated, oocytes were clamped at −60 mV. Continuous recordings at 500 Hz were taken during the application of a panel of agonists or antagonists. Glass electrodes were pulled on a P1000 Micropipette Puller (Sutter), with a resistance ranging from 0.7–2 MΩ. Electrodes contained Ag|AgCl wires and backfilled with a 1.5 M KCl and 1 M acetic mixture. Data were recorded using the RoboCyte2 control software, or with WinWCP for manual recordings, and filtered at 10 Hz.

Peak current for each dose was normalized to the oocyte maximum current using a custom-built python script (40). For dose–response applications a protocol with 10-s agonist application pulses, with 60 s of ND96 wash in between each dose was used. Using different batches of oocytes, the data were gathered over at least two occasions. After normalization, the data were imported into Graphpad (Prism) and fitted to Hill equation (either three or four parameter nonlinear) to obtain the highest degree of fit and to calculate the EC_50_. The EC_50_ dose of the primary agonist was used for antagonist dose–response measurements as well as for ion selectivity recordings. Antagonist dose–response protocols used 10-s applications of agonist with antagonist present, followed by 60 s of ND96 washes between doses. The agonist concentrations remained constant. IC_50_ values for each antagonist were calculated using a second custom-built python script (40). After normalization, the data were fitted to a nonlinear Hill equation (either a three or four parameter) in Graphpad (Prism) to obtain the highest degree of fit and calculate the IC_50_.

A voltage ramp protocol from −80 mV to +60 mV (20 mV/s) was used for ion selectivity measurements, in the presence of the primary agonist in three different solutions: ND96, NMDG (Na^+^ free), and Na Gluconate (low Cl^−^) solutions. The data were normalized to maximum current (I_max)_, and the reversal potential (ΔE_Rev_) was calculated using a custom-built python script (40). Individual values, or mean, SD and n, for each construct was imported in GraphPad for further plotting and statistical analysis. Statistically significant differences in ΔE_Rev_ were calculated in GraphPad using a two-way ANOVA with Tukey’s correction for multiple comparisons. A representative normalized trace for each construct was also generated in Graphpad.

### Confocal and Cell ID.

Worms were mounted onto 2% agarose (in M9) pad and immobilized with 75 mM NaAz (in M9). A 63× water immersion lens on a Leica SP8 or STED or a 40× oil immersion objective on a Zeiss LSM780 was used to acquire image stacks; these stacks were collapsed and max projections of z-stack images were generated in Fiji/Image J. Individual neurons were identified by cell shape, position, or by crossing the reporter lines with the multicolour reference worm NeuroPAL (41).

### Metabolite Extraction.

Worm samples frozen in liquid nitrogen were lyophilized to dryness. Dried worm pellets were homogenized by grinding using steel balls (2.5-mm balls, SPEX sample prep miniG 1600) at 1,200 RPM for 3 min in 1-min pulses and then extracted with 20 mL methanol for 24 h. The extracts were dried with a SpeedVac Vacuum Concentrator (ThermoFisher Scientific) and resuspended in 500 μL MeOH, transferred to 1.7 mL Eppendorf tubes, and centrifuged at 18,000 *g* for 10 min at 4°C. Concentrated extracts were transferred to HPLC vials for HPLC–HRMS analysis. For bacterial metabolite extraction, bacteria were grown in either regular LB media or M9 media supplemented with 0.2% glucose as well as 1 μg/ml thiamine.

### Mass Spectrometric Analysis.

High-resolution LC−MS analysis was performed on a ThermoFisher Scientific Vanquish Horizon UHPLC System coupled with a Thermo Q Exactive HF hybrid quadrupole-orbitrap high-resolution mass spectrometer equipped with a HESI ion source. 1 μL extract was injected and separated using a water-acetonitrile gradient on an Agilent Zorbax Hilic Plus column (150 mm × 2.1 mm, particle size 1.8 μm) maintained at 40°C with a flow rate 0.3 mL/min. HPLC grade solvents were purchased from Fisher Scientific. Solvent A: 0.1% formic acid in water; Solvent B: 0.1% formic acid in acetonitrile. Analytes were separated using a gradient profile as follows: 2 min (95% B) → 20 min (50% B) → 22 min (10% B) → 25 min (10% B) → 27 min (95% B) →30 min (95% B). Mass spectrometer parameters: spray voltage 3.0 kV, capillary temperature 380°C, prober heater temperature 300°C; sheath, auxiliary, and spare gas 60, 20, and 2, respectively; S-lens RF level 50, resolution 240,000 at m/z 200, AGC target 3 × 106. The instrument was calibrated with positive and negative ion calibration solutions (ThermoFisher). Each sample was analyzed in positive and negative modes with m/z range 100–700. As a reference for betaine, betaine hydrochloride was purchased from Sigma-Aldrich. HRMS (ESI) m/z: calculated for C_5_H_12_NO_2_^+^, [M + H]^+^: 118.0863, found: 118.0860.

### Betaine Vesicular Transporter Assay.

Previously characterized *C. elegans* vesicular transporter genes (*unc-17, cat-1*), three uncharacterized *C. elegans* solute carrier genes (*T28F3.4, ZK54.1a*, and *ZK682.2*), and the human betaine/GABA vesicular transporter, bgt1 (SLC6A12) were expressed in *Xenopus* oocytes. After 3-d incubation at 16°C, the oocytes were incubated in 100 nM ^3^H-Betaine (ARC, USA) for 10 min, followed by four washes with ND96. Each oocyte (6–10/gene) was lysed in a 24-well plate using 200 μl 1% SDS. 3 ml scintillation liquid (PerkinElmer) was added to each sample and radioactivity was determined using a Tri Carb 4910TR liquid scintillator (PerkinElmer).

### Food Leaving Assays.

Assays were carried out as described in ref. 42. Briefly, 20 1-d adults were placed onto 55-mm low-peptone agar assay plates seeded the day before with a 20 μL OP50 food patch in the center. Fifteen-min videos were captured with DinoLite cameras (DinoLite, model no. AM7915MZTL) at the time points: 1 h, 3 h, 6 h, and 9 h after placing worms onto the assay plates. Worm skeletons and trajectories were analyzed using tierpsy (43), and the number of food leaving events was counted and expressed as food leaving probability. A leaving event was characterized by the full length of the worm’s body leaving the food patch. Food leaving probability was calculated as follows: Number of leaving events/the number of worms on the food patch at the start of the video/length of the video (min).

### Chemotaxis Assays.

Assays were carried out using the quadrant method as described in ref. 44. Briefly, 1-d adults (synchronized by placing L4 worms on culture plates overnight, 4 d before the day of the assay) were washed 3 times in M9 and transferred onto 55-mm CTX assay plates prepared with two test and two control substances, each mixed 1:1 with 1 M NaAzide, totalling 2 μL per dot. 2-nonanone and diacetyl were diluted in 100% ethanol, betaine was dissolved in M9, in each case the diluent was used as the control substance. After 1 h, the number of worms in each quadrant was counted, and the chemotaxis index calculated as follows: Chemotaxis Index = (No. of worms in Test 1 + No. of worms in Test 2) − (No. of worms in Test 1 + No. of worms in Test 2)/Total No. of worms. Worms that did not leave the center of the plate were not counted, and only plates with a minimum of 50 worms were used in the analysis.

### 2-Nonanone-Induced Food Leaving Assays.

Assays were carried out as described in ref. 26. Briefly, 20 1-d adults were placed onto 55-mm low-peptone assay plates, seeded the previous day with 20 μL OP50. After 1 h, and during video recording, a 1-μL dot of 100% 2-nonanone was placed directly next to, but not touching the food patch. The number of worms remaining on the food patch at 1-min intervals was recorded for 10 min, then again at 15 min and calculated as a percentage of the total worms on the food before 2-nonanone addition.

### Dispersal Assays.

1-d adult worms were synchronized by placing L4 worms on culture plates overnight, 4 d before the day of the assay. Worms were washed four times in M9 buffer to remove any bacteria and a densely packed 8 μL volume of worms was pipetted into the center of the 55-mm CTX agar assay plate. Once the M9 from the transfer dried, worms were left on the assay plates for 1 h at which photos were taken of each plate. Images were processed in Image J to remove background using thresholding, IntDen (integrated density) of a defined central zone, and the whole plate was then calculated. The zone density ratio was then calculated as follows: zone density ratio = (IntDen central zone/central zone area)/(IntDen whole plate/whole plate area).

### Global Search Assay.

1-d adult worms either fed with OP50 or *Bet*A bacteria grown in LB or M9 minimal media (supplemented with 0.2% glucose and 1 μg/ml thiamine), were synchronized by picking L4 worms the day before the assay. Adult worms were briefly transferred onto an empty NGM plate and allowed to crawl for at least 30 s to remove any excess bacteria, 10–20 worms were then placed in the center of an empty 8-cm low-peptone NGM assay plate. After a 30-s rest period a 0–5-min and 40–45-min video was captured. Prior to the 40–45-min timepoint, worms were carefully moved back into the center of the assay plate and given an additional 30-s rest period before the start of the video. Videos were captured using a Ximea MQ042RG-CM camera with a modular lens by Navitar (6.5X Zoom, (1-60135), 0.5X standard adapter (1-60439), 0.25X lens attachment (1-6044), 1.0X standard adapter (1-6015), and backlit with a 625-nm LED panel (Vision Light Tech, ELF-400X400RD), achieving a field of view of 5 cm^2^ at 25 fps. Worm skeletons and trajectories were calculated using tierpsy (43), where necessary trajectories for each worm were manually joined within the tierpsy GUI. A custom python pipeline was generated in which individual worm tracks of at least 30 s were filtered with a 50-frame rolling mean to remove normal sinusoidal movement and any gaps shorter than 5 s were linearly interpolated. Curvature was calculated for each frame and any curvature events above 0.25 were classified as a “reorientation”, which was then used to create the metric: reorientations per worm per minute.

### Worm Tracking.

Four 1-d adult worms were placed into the center of the food patch on 55-mm NGM plates seeded with 4 μL OP50 with or without 10 mM betaine 2 h before. Fifteen-min videos were taken with DinoLite (DinoLite, model no. AM7915MZTL) cameras at 40X magnification and worm behavior analyzed with tierpsy (43). Where necessary, trajectories for each worm were manually joined within the tierpsy GUI, the summary features for each trajectory were then exported, and statistically significant differences were calculated for four basic features: motion_mode_forward_fraction, motion_mode_backward_fraction, speed_10^th^, and speed_50^th^.

### Data Analysis.

Unless otherwise specified data were imported into GraphPad for further analysis and plotting. Unless otherwise specified, a one-way ANOVA was used to calculate significant differences, using the Bonferroni (food leaving & dispersal), Kruskal–Wallis test (reorientation data) or Tukey’s (food leaving time course, chemotaxis) correction for multiple comparisons. *P < 0.05, **P < 0.005, ***P < 0.001, ****P < 0.0001. Python scripts for TEVC analysis and tracking analysis are deposited on GitHub at hiris25/TEVC-analysis-scripts and hiris25/Tierpsy-Tracking-Analysis, respectively. Data used for analyzing TEVC experiments are available upon request from the Lead Contact.

## Supplementary Material

Appendix 01 (PDF)

## Data Availability

Python scripts for TEVC analysis, Python scripts for tracking analysis, and associated data have been deposited in Github (hiris25/TEVC-analysis-scripts; hiris25/Tierpsy-Tracking-Analysis).
