# Supplementary material for: Neuronally produced betaine acts via a ligand-gated ion channel to control behavioral states
Source: Proc Natl Acad Sci U S A. 2022 Nov 21;119(48):e2201783119. doi: 10.1073/pnas.2201783119 (PMC9860315; doi:10.1073/pnas.2201783119)
Supplement: Supplementary file 1 — Appendix 01 (PDF) [file pnas.2201783119.sapp.pdf]

Figure S1

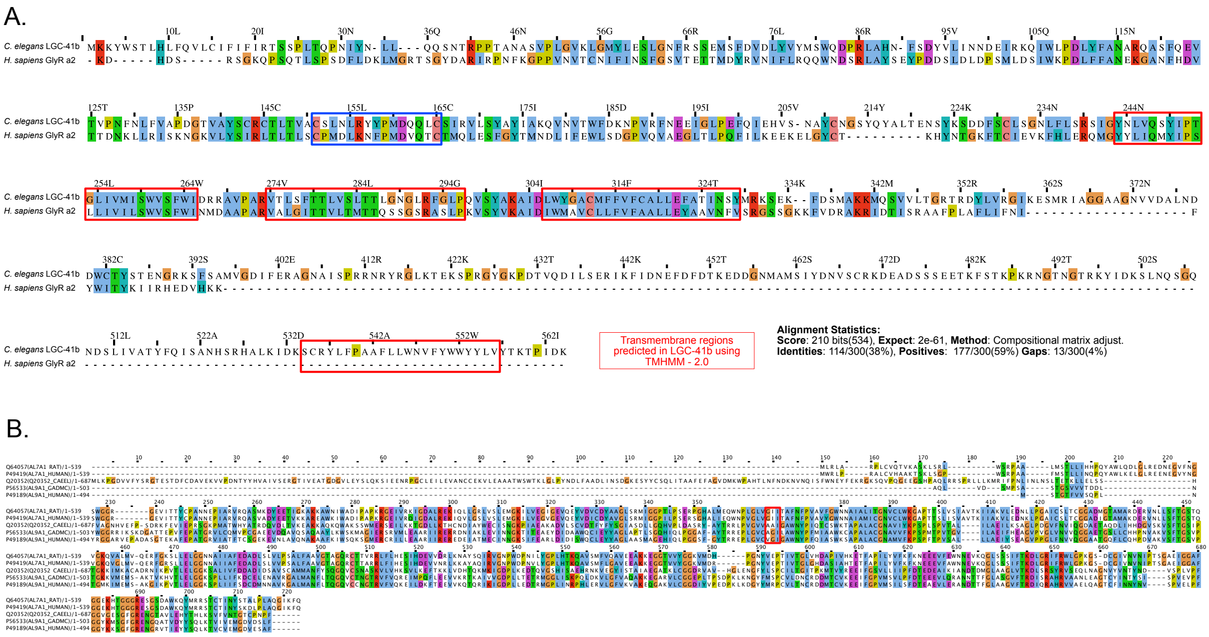

Supplementary Figure 1. **Alignments of *C. elegans* LCG-41 and ALH-11 with homologs from other species.** (**A-B**) Full length alignments generated with CLUSTAL omega. (**A**) Alignment of *C. elegans* LGC-41 and human GLYRA2. Red boxes show location of transmembrane domains in LGC-41 predicted by TMHMM2.0. Blue box highlights conserved cys-loop. (**B**) Alignment of *C. elegans* ALH-11 with close homologs from rat, human and cod, uniport IDs given. Red box shows location of *alh-11(lj118)* mutation.

Figure S2

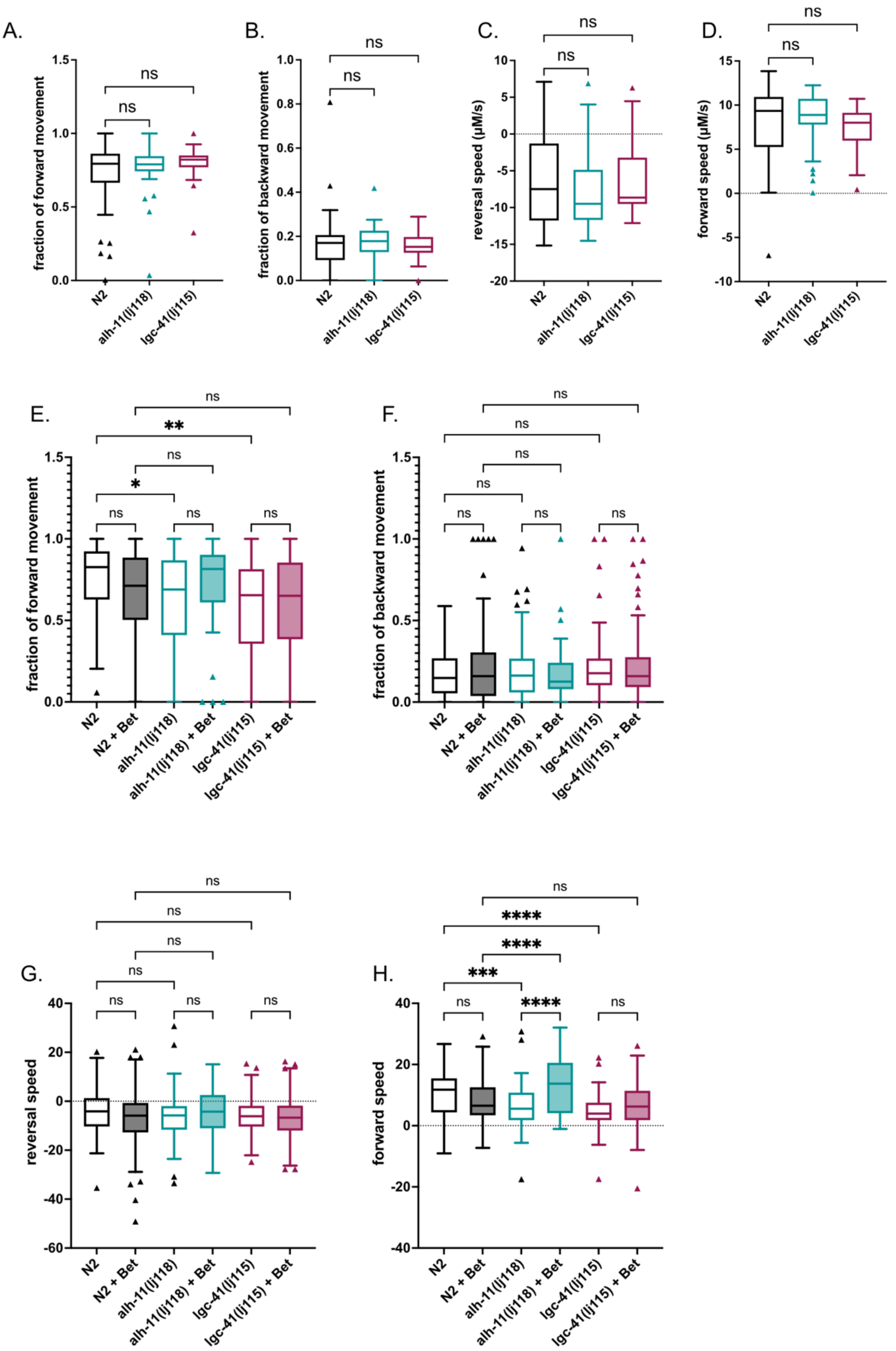

<u>Supplementary Figure 2.</u> **Basic locomotion features of N2 and *lgc-41* and *alh-11* mutant worms on food, off food and on food supplemented with betaine.** Basic locomotion features of N2 (wild-type), *alh-11(lj118)* and *lgc-41(lj115)* animals, shown as Tukey's box plots, (**A-**

D) in the first 5 min off food, (**E-H**) over a 15 min period on food (OP50), in the presence or absence of 10 mM betaine. Statistical significance calculated by one-way ANOVA with Sidak’s correction for multiple comparisons, *=P<0.05, **=P<0.01, ***=P<0.005, ****=P<0.001, ns=not significant. (**A-D**) N=39-53 animals (**E-H**) N=62-159 animals. Features are calculated using tierpsy summary function and have been renamed for simplicity as follows: fraction of forward movement = ‘motion_mode_forward_fraction’, fraction of reverse movement = ‘‘motion_mode_backward_fraction’, reversal speed = ‘speed_10^{th}’, forward speed = ‘speed_50^{th}’.

Figure S3

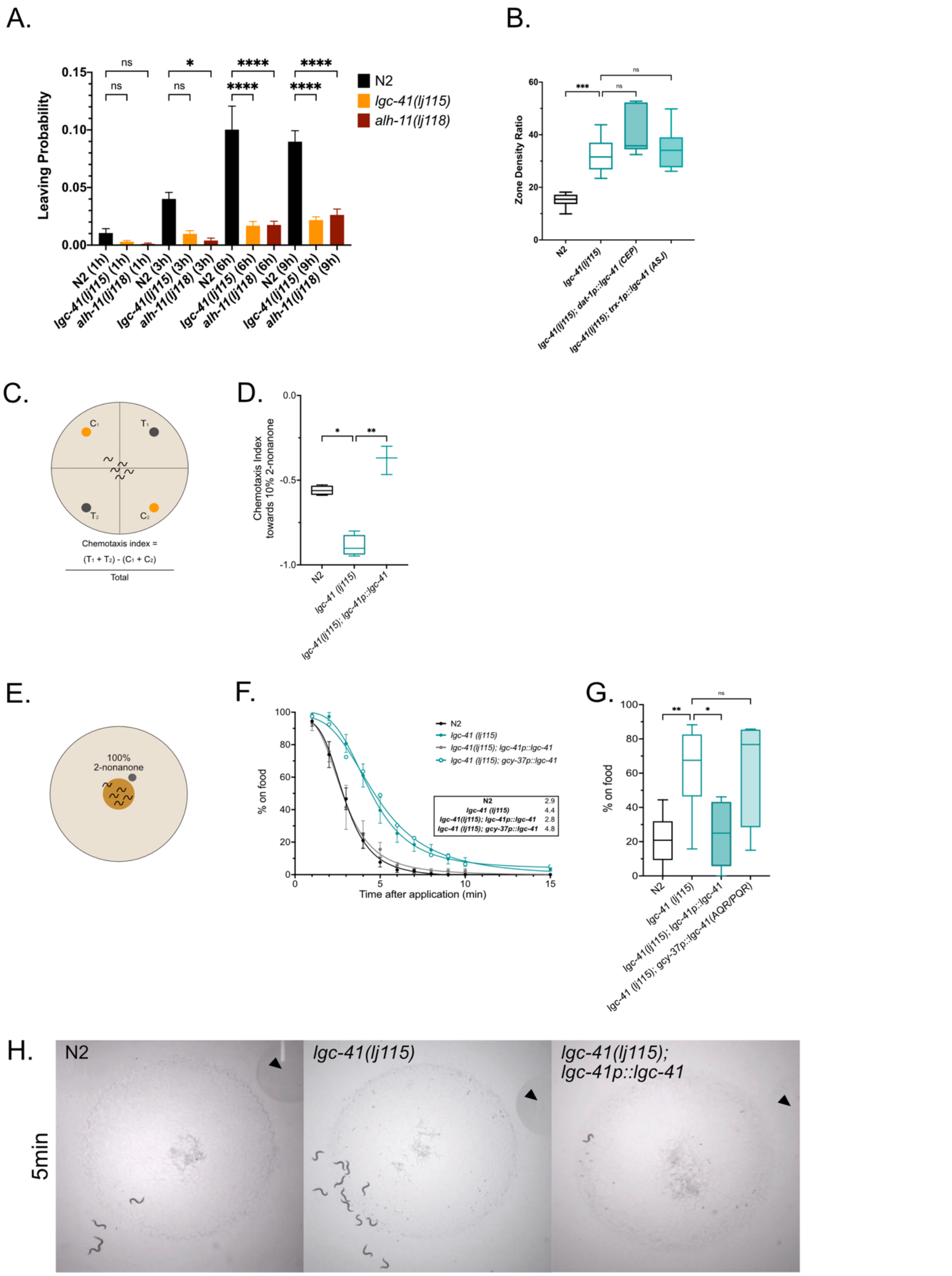

Supplementary Figure 3. ***lgc-41* mutant worms display defective 2-nonanone induced food leaving behaviour.** Behavioural responses of N2 (wild-type), *lgc-41(lj115)*, *lgc-41(lj115);*

lgc-41p::lgc-41, lgc-41(lj115); gcy-37p(AQR/PQR)::lgc-41, lgc-41(lj115); dat-1p(CEP)::lgc-41, lgc-41(lj115); trx-1p(ASJ)::lgc-41and alh-11(lj118) worms. (**A**) Food leaving probability time course, n=5 plates for each genotype and each time point. Significance calculated by two-way ANOVA with Tukey's correction for multiple comparisons. * $P<0.05$, **** $P<0.0001$. (**B**) Box plot of dispersal propensity of N2, *lgc-41(lj115), dat-1p(CEP)::lgc-41* and *lgc-41(lj115); trx-1p(ASJ)::lgc-41* worms, in which central zone density is calculated and plotted. N=6-8 plates per genotype. Tukey's blot plots and one-way ANOVA with Tukey correction for multiple comparisons, ***=$P<0.001$. (**C**) Schematic representation of chemotaxis experiment, (**D**) box plot of chemotaxis index towards 10% 2-nonanone. N=3-5 plates per genotype. (**E**) Schematic representation of the experimental design of 100% 2-nonanone induced food leaving, the graph (**F**) displays percentage of worms on food each minute after 2-nonanone application for 15 min, curves fit with a four-parameter variable slope, insert gives time in minutes when 50% of worms have left the food patch. N=5-12 plates per genotype, error bars represent SEM. (**G**) Box plot of percentage of worms on the food patch 4 min after 2-nonanone application. N=5-12 plates per genotype. (**H**) Representative images of N2, *lgc-41* mutant and *lgc-41* rescue worms 5 min after 2-nonanone application, black arrow indicate 2-nonanone droplet.

Figure S4

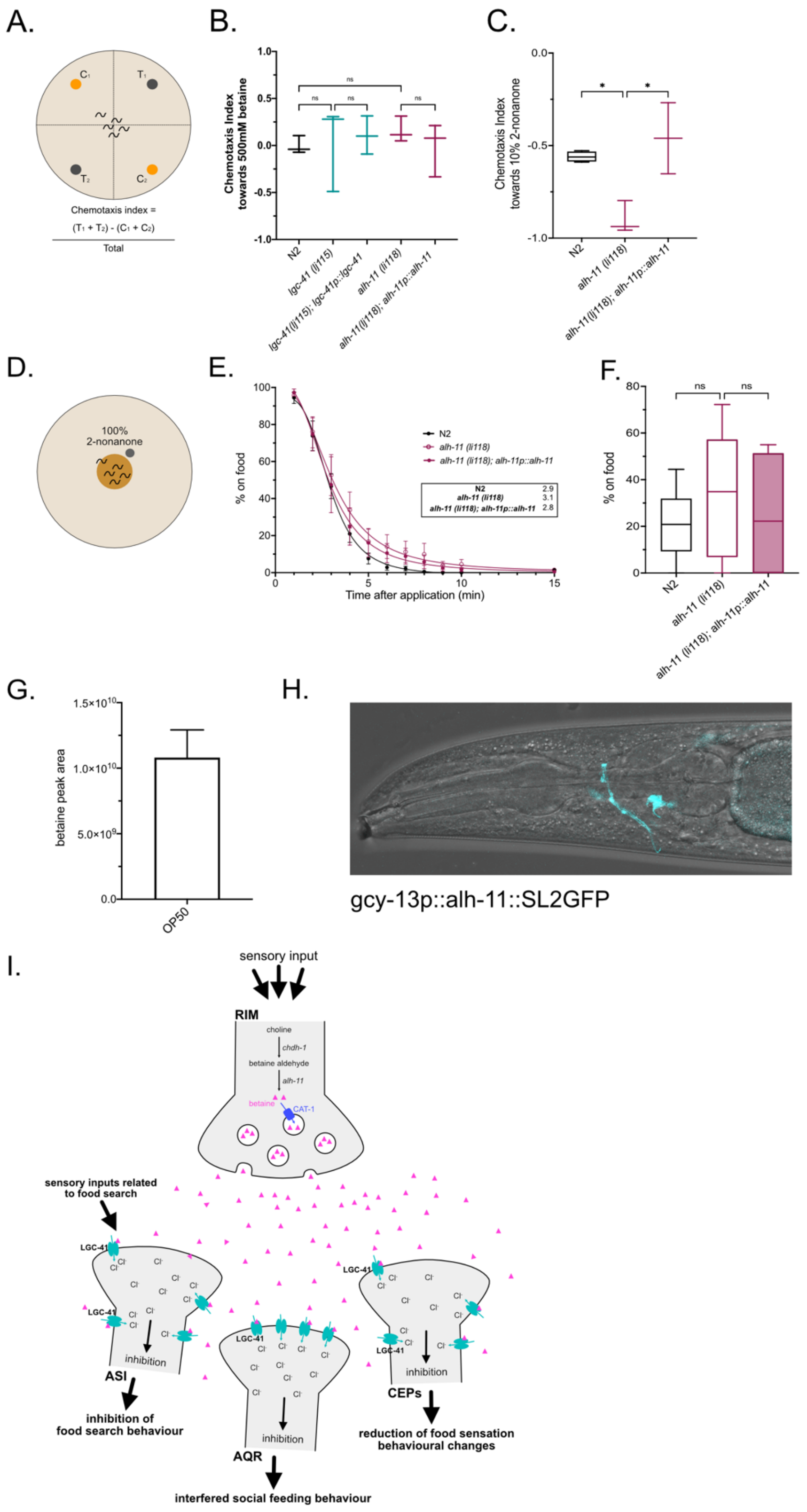

Supplementary Figure 4. ***lgc-41* and *alh-11* mutant worms display normal betaine chemotaxis, *alh-11* worms also display normal 2-nonanone induced food leaving**

behaviour. Behavioural responses of N2 (wild-type), *lgc-41(lj115)*, *lgc-41(lj115); lgc-41p::lgc-41, alh-11(lj118)* and *alh-11(lj118);alh-11p::alh-11* worms. (**A**) Schematic representation of chemotaxis experiment, (**B**) Chemotaxis towards or away from 500 mM betaine in wildtype (N2) or *lgc-41(lj115)*, *alh-11(lj118)* mutants or genomic rescue lines. N=3-5 per genotype, significance calculated by one-way ANOVA with Tukey's correction for multiple comparisons. (**C**) box plot of chemotaxis index towards 10% 2-nonanone. N=3-5 plates per genotype, * P<0.05. (**D**) Schematic representation of the experimental design of 100% 2-nonanone induced food leaving, the graph (**E**) displays percentage of worms on food each minute after 2-nonanone application for 15 min, curves fit with a four-parameter variable slope, insert gives time in minuets when 50% of worms have left the food patch. N=5-12 plates per genotype, error bars represent SEM. (**F**) Box plot of percentage of worms on the food patch 4 min after 2-nonanone application. N=5-12 plates per genotype. (**G**) Mass spectrometry analysis of betaine content in *E.coli (*OP50). The data shows peak area for betaine. Error bars represent SEM of 5 samples. (**H**) Fluorescent reporter image of RIM specific expression of *alh-11* under the control of *gcy-13p*. (**I**) Graphical cartoon outlining the suggested betaine and LGC-41 mechanism.

Figure S5

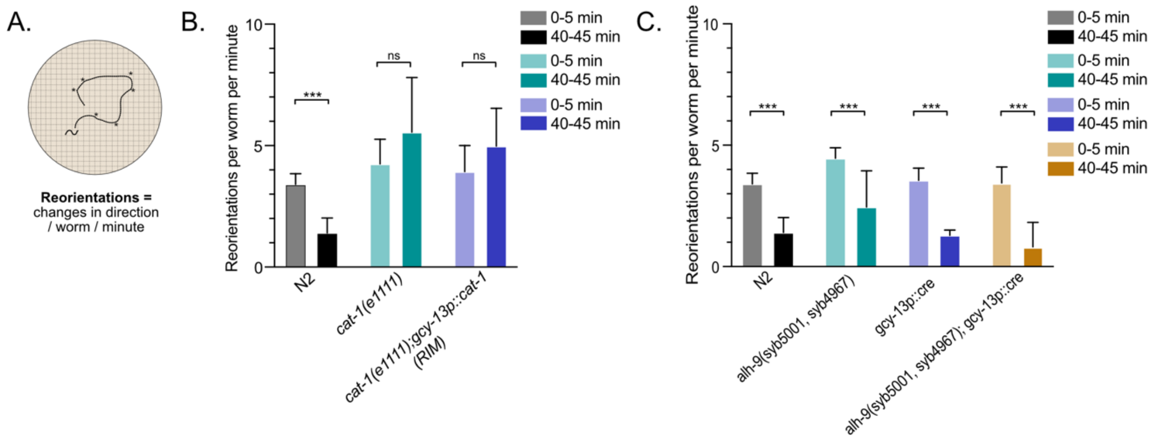

Supplementary Figure 5. ***cat-1* but not *alh-9* RIM mutants are defective in global food search.** (**A**) Schematic describing the principle of the reorientation experiment. (**B**) Worm reorientation events off food at two different time points show that *cat-1* mutant worms are defective in switching between local and global food search. The defect cannot be rescued by expressing *cat-1* in the RIM neurons. (**C**) Reorientation events in a cre-induced *alh-9* CRISPR mutant line shows no effect on the food search behaviour upon expression of cre in the RIM neurons. (**B, C**) The data is presented as median values with 95% CI, a Kruskal-Wallis test was used for calculation significant difference between the timepoints and groups. * P<0.05, ** P<0.005, *** P<0.0005.

Table S1: Strain list

Strain No.	Description
AQ4633	*alh-11(lj118) (bc 3x)*
AQ4618	*lgc-41(lj115) (bc 4x)*
AQ4658	*lgc-41(lj119) (bc 4x)*
AQ4888	*liEx1479[alh-9p::alh-9::SL2GFP; unc-122p:RFP] on OH15262*
AQ4913	*ljEx1489[chdh-1p::chdh-1::SL2 GFP; unc-122p::RFP] on OH15262*
AQ4550	*him-5(e1490); ljEx1305[alh-11p(3kb)::alh-11::SL2-mkate2; unc-122p::gfp]*
AQ4525	*him-5(e1490); ljEx1297[alh-9p(1.5kb)::alh-9::SL2-mKate2; unc-122p::gfp]*
AQ4523	*him-5(e1490); ljEx1295[chdh-1p(2kb)::chdh-1::SL2-mKate2; unc-122p::gfp]*
AQ4404	*him-5(e1490); ljEx1250[lgc-41p(2kb)::lgc-41::SL2-mKate2; unc-122p::gfp]*
AQ4391	*ljEx1244[lgc-41p(2kb)::mKate2::gpd-23'UTR; unc-122p::gfp]*
OH15262	*"NeuroPAL" otEx7057*
AQ5152	*alh-11(lj118); ljEx1595[alh-11p(3kb)::alh-11::SL2GFP; unc-122p::RFP]*
AQ5177	*alh-11(lj118); ljEx1603[gcy-13p::alh-11::SL2GFP; unc-122p::RFP]*
AQ5135	*lgc-41(lj115); ljEx1584[gcy-37p::lgc-41::SL2mKate; unc-122p::gfp]*
AQ5088	*lgc-41(lj115); ljEx1576[daf7p(4.5kb)::lgc-41::Sl2mKate; unc-122p::gfp]*
AQ5224	*lgc-41(lj115); ljEx1619[dat-1p::lgc-41::Sl2mKate; unc-122p::gfp]*
AQ5225	*lgc-41(lj115); ljEx1619[trx-1p::lgc-41::Sl2mKate; unc-122p::gfp]*
AQ5241	*alh-9(syb5001, syb4967); ljEx1643[gcy-13p::cre::sl2GFP, unc-122p:RFP]*
AQ5242	*ljEx1644[gcy-13p::cre::sl2GFP, unc-122p:RFP]*
AQ5235	*alh-9(syb5001, syb4967)*
AQ5277	*cat-1(e1111); ljEx1649[gcy-13p::cat-1 cDNA::sl2GFP, unc-122p:RFP]*
AQ0149	*cat-1(e1111)*

Note:
chdh-1 previously called *C34C6.4*
bc: backcrossed

Bacterial strain list:

Strain name	Aquired from
OP50	CGC
betA(JW0303)	Horizon Discovery Ltd
K12(BW25113)	Horizon Discovery Ltd

Table S2: CRISPR alleles

Allele	***N2 sequence***	***Mutant sequence***
lgc-41 (lj115) frameshift	Tgatttgtactttgcgaatgcaaggcaagcaagctttcaagaggttactgttcccaattttaatctgtttgttgcaccagatggtactgtcgcatactcctgtagatgtacacttacggtggcttgtagtttgaacctaaggtaaaaaaaaagtaaataaactgaatg	TGATTTGTACTTTGCGAATGCAAGGCAAGCAAGCTTTCAAGAGGTTACTGTTCCCAATTTTAATCTGTTTGTTGCACCAGATGGTACTGTCGCATACTCCTGTAGATGTACACTTAGGCTTGTAGTTTGAACCTAAGGTAAA

	ttaatttcgcaattttagatactacccaatggat<br>caacagctttgtt	AAAAAAGTAAATAAACTGAATGTTAATT<br>TCGCAATTTTAGATACTACCCAATGGATC<br>AACAGCTTTGTT
alh-11 (lj118) frameshift	ggtgacagcgggctgggcttatatatgacagca<br>tttccacaggccagtgctggtgcagttttccagg<br>agcatgtttgaattggatagttccaggcaccgat<br>ggcggcgaccactccaacaggaagacggcgtg<br>tataggcgtaacgagaagcatcgagtggaacg<br>tgttgaccgagaaggtcagatgctagattttgg<br>agatcagg	ggtgacagcgggctgggcttatatatgacagcatttc<br>cacaggccagtgctggtgcagttttccaggagcatgt<br>ttgaattggatagttccaggcaccgacgaccactcca<br>acaggaagacggcgtgtataggcgtaacgagaagc<br>atcgagtggaacgtgttgaccgagaaggtcagatgc<br>tagattttggagatcagg
lgc-41 (lj119) insertion	aaacacttttaaactttttcagcggttcctgctcg<br>cgtcacattaagtttcaccacactggtctctctta<br>ccactctggtaagtttttgagttacttgggttgac<br>gttcagccgatgcgtcttctctgcaagaatgaca<br>tatcactttttttagggaaacggacttcgatttgg<br>acttccacaagtgagctatgcaaaagccatcga<br>cttgtggtacggaggtatcaaagaacaaatcca<br>cccatttgggaatacacattattcaacttctttca<br>gcgtgtatgttcttcgtcttttgtgcacttttggaa<br>tttgcaactataaatagctacatgcgaaagtcg<br>gaaaaatttgatagtatggcaaagaaaatgca<br>aagtgtggtactcactggaagaagtaagatatg<br>gcaatagactgcaaatccaaaaatttcagattg<br>catggttatattcactgatcgtagtatctctatttt<br>tcagttcccccatcttacaatccatttactctaat<br>ttacagacttttgcacgacgcacgatccaaata<br>gtttcttgcactcattttttttgtttccacttctattt<br>ctatcacatggaagattgaagtatgcaccgata<br>ccgtttaattttgcatctctcaatccgtacaatta<br>atgtactaagttaggtcaaatctgaaattccag<br>caagggactatttggttcgcggcatcaaagaga<br>gcatgcggattgcaggaggagcagccggaaac<br>gttgtggacgcactcaacgacgattggtgtacct<br>actccacagagaacggacgtaagagcttctcc<br>gcaatggtcggagacatttttgaaagagccggc<br>aatgcgatttctccccgtagaaacagatacaga<br>ggtgtagatcagtgtggacatttccttttaatca<br>cactaaaatccgtgttttggttttgatttggtttgc<br>ctggagacgtctagaaaacatgttaaattccgtt<br>agagtaaacatagtcaatttggaaaaacagaa<br>atagtggaagttagattttatttcttgaggaata<br>aacaattaatgtataagaagcaattctaaacat<br>acacgtaaacctttattttgttcttttgcaaccca<br>gcacaattgtttcatataggaaactcaaagtac<br>caagtacccaaaagtttacttgactccttcccaa<br>ccccctcacgttctccatatccatcagtgaaact<br>atcgtttatctatctgtaactgtgttgttactttc	GGttcagccgatgcgtcttctctgcaagaatgacat<br>atcactttttttagggaaacggacttcgatttggacttc<br>cacaagtgagctatgcaaaagccatcgacttgtggt<br>acggaggtatcaaagaacaaatccacccatttggga<br>atacacattattcaacttctttcagcgtgtatgttcttc<br>gtcttttgtgcacttttggaatttgcaactataaatagc<br>tacatgcgaaagtcggaaaaatttgatagtatggca<br>aagaaaatgcaaagtgtggtactcactggaagaagt<br>aagatatggcaatagactgcaaatccaaaaatttca<br>gattgcatggttatattcactgatcgtagtatctctatt<br>tttcagttcccccatcttacaatccatttactctaattt<br>acagacttttgcacgacgcacgatccaaatagtttctt<br>gcactcattttttttgtttccacttctatttctatcacatg<br>gaagattgaagtatgcaccgataccgtttaattttgc<br>atctctcaatccgtacaattaatgtactaagttaggtc<br>aaatctgaaattccagcaagggactatttggttcgcg<br>gcatcaaagagagcatgcggattgcaggagCagca<br>ATGagtaaaggagaagaattgttcactggagttgtc<br>ccaatcctcgtcgagctcgacggagacgtcaacgga<br>cacaagttctccgtctccggagagggagagggagac<br>gccacctacggaaagctcaccctcaagttcatctgca<br>ccaccggaaagctcccagtcccatggccaaccctcgt<br>caccaccttctgctacggagtccaatgcttctcccgtt<br>acccagaccacatgaagcgtcacgacttcttcaagtc<br>cgccatgccagagggatacgtccaagagcgtaccat<br>cttcttcaaggtaagtttaaacatatatatactaacta<br>ctgattatttaaattttcaggacgacggaaactacaa<br>gacccgtgccgaggtcaagttcgagggagacaccct<br>cgtcaaccgtatcgagctcaaggtaagtttaaacagt<br>tcggtactaactaaccatacatatttaaattttcaggg<br>aatcgacttcaaggaggacggaaacatcctcggaca<br>caagctcgagtacaactacaactcccacaacgtcta<br>catcatggccgacaagcaaaagaacggaatcaagg<br>tcaacttcaaggtaagtttaaacatgattttactaact<br>aactaatctgatttaaattttcagatccgtcacaacat<br>cgaggacggatccgtccaactcgccgaccactacca<br>acaaaacaccccaatcggagacggaccagtcctcct

	gttcaacatcatctatccggatgcagtttccaaa cgctccccaaacacactatttagttgttcgtaac ctcggtttacctgaataccatccactgtcatcaa tattagcaaaactatcactctagttttctaatccc ttttattagcgtactgg	cccagacaaccactacctctccacccaatccgccctc tccaaggacccaaacgagaagcgtgaccacatggtc ctcctcgagttcgtcaccgccgccggaatcacccacg gaatggacgagctctacaaggtaagtttaaaataac ttcgtatagcatacattatacgaagttattttcagggt ggcagcggaggtaccggcggtagtggaggcACGg ccggaaacgttgtggacgcactcaacgacgattggt gtacctactccacagagaacggacgtaagagcttct ccgcaatggtcggagacatttttgaaagagccggca atgcgatttctccccgtagaaacagatacagaggtgt agatcagtgtggacatttccttttaatcacactaaaat ccgtgttttggttttgatttggtttgcctggagacgtct agaaaacatgttaaattccgttagagtaaacatagtc aatttggaaaaacagaaatagtggaagttagatttt atttcttgaggaataaacaattaatgtataagaagca attctaaacatacacgtaaacctttattttgttcttttg caacccagcacaattgtttcatataggaaactcaaa gtaccaagtacccaaaagtttacttgactccttccca accccctcacg

Flanking sequence
Added/removed sequence
